# Ruptured pseudoaneurysm of the thoracic aorta mimicking lung cancer: A case report

**DOI:** 10.1111/1759-7714.13783

**Published:** 2021-01-09

**Authors:** Yutaka Takahara, Kazuaki Nishiki, Keisuke Nakase, Shiro Mizuno

**Affiliations:** ^1^ Department of Respiratory Medicine Kanazawa Medical University Kahoku‐gun Japan

**Keywords:** Lung cancer, pseudoaneurysm of thoracic aorta, ruptured aneurysm

## Abstract

A 70‐year‐old woman was admitted to a local hospital with a five‐day history of back pain. She had been referred to our hospital after an abnormal chest shadow was identified on chest X‐ray. Chest computed tomography (CT) revealed an anterior mediastinal mass in the upper lobe of the left lung. Her general condition was good. She was diagnosed with lung cancer, and examination was planned. However, respiratory failure rapidly worsened on hospital day 2, and a ruptured pseudoaneurysm of the thoracic aorta (PTA) was diagnosed from contrast‐enhanced CT. Emergency thoracic endovascular aortic repair was successfully performed, and her postoperative course was uneventful. The hemodynamics of the PTA were stable in the case of this patient, but the risk of rupture is extremely high and frequently fatal. PTA should therefore be included among the differential diagnoses of mediastinal tumor.

**Key points:**

**Significant findings of the study:**

Pseudoaneurysm of the thoracic aorta (PTA) may present on imaging findings that resemble lung cancer.

**What this study adds:**

PTA should be included among the differential diagnoses of mediastinal tumor. Clinicians therefore need to be familiar with the imaging findings of PTA.

## Graphical abstract

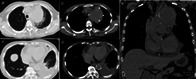

PTA shows stable hemodynamics, but the risk of rupture is extremely high and frequently fatal. To assist in the differential diagnoses, clinicians need to be familiar with imaging findings of PTA.

## Introduction

Diagnosis of thoracic aortic aneurysm from computed tomography (CT) is considered relatively easy, but some reports have previously described cases that have been difficult to distinguish from mediastinal tumor.^1^, ^2^, ^3^ In particular, rupture of an aortic aneurysm requires urgent treatment, and clinical identification is thus extremely important. Aneurysm rupture usually presents with rapid onset of hemorrhagic shock. However, we encountered a case with pseudoaneurysm in which the hemodynamics were stable at the first visit. Distinguishing this pathology from lung cancer was difficult based on plain CT alone. Here, we report the case of a patient who survived rupture of a pseudoaneurysm of the thoracic aorta (PTA) after being diagnosed with lung cancer at the first visit.

### Case report

**Figure 1:**
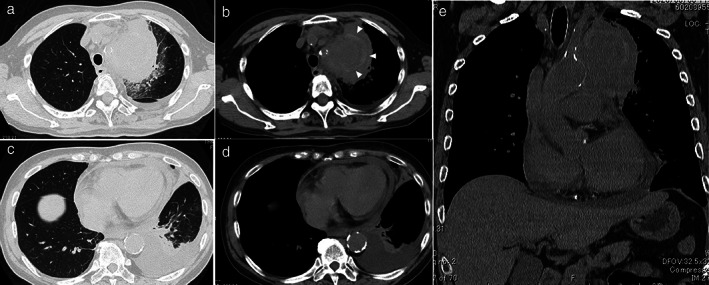
Chest computed tomography (CT) on admission. (**a**, **c**) Lung windows. (**b**, **d**, **e**) Soft‐tissue windows. Chest CT shows a tumor shadow in the upper lobe of the left lung, pleural effusion, and pericardial effusion. A region of ground‐glass attenuation is apparent around the tumor. A linear region of high attenuation (arrowheads) is apparent inside the tumor.

A 70‐year‐old Japanese woman was admitted to a local hospital with a five‐day history of back pain. She was referred to our hospital after an abnormal chest shadow was identified on chest X‐ray. She was a current smoker with a 25‐pack per year history of smoking. She was taking telmisartan (20 mg/day), bisoprolol (5 mg/day), alogliptin‐pioglitazone (25–15 mg/day), glimepiride (0.5 mg/day) and fluvastatin sodium (20 mg/day) for pre‐existing conditions such as hypertension, type 2 diabetes, and hyperlipidemia. On admission, she expectorated a small volume of bloody sputum, but had no history of trauma. Physical examination on admission revealed blood pressure, 138/88 mmHg; heart rate, 88 beats/minute; temperature, 36.6°C; and percutaneous oxygen saturation in room air, 97%. Cardiovascular examination revealed no abnormalities and breath sounds were clear. Laboratory examinations (Table 1) showed white cell count, 15 410/μL; C‐reactive protein (CRP), 28.92 mg/dL; hemoglobin A1c, 9.1%; and glucose, 305 mg/dL. Chest CT (Fig 1) revealed an anterior mediastinal mass in the upper lobe of the left lung. Based on these findings, locally advanced lung cancer with mediastinal invasion was diagnosed.

**Table 1:** Laboratory data on admission

Hematology			Biochemistry			Serology		
WBC	15 410	/μL	AST	26	U/L	CRP	28.92	mg/dl
Neut.	88.7	%	ALT	19	U/L			
Lym.	3.8	%	γ‐GTP	18	U/L			
Eos.	0.2	%	LDH	209	U/L			
RBC	359 × 10^4^	/μL	BUN	22	mg/dL			
Ht	33.1	%	CRE	0.95	mg/dL			
Hb	11.3	g/dl	Alb	2.6	g/dl			
PLT	25.8 × 10^4^	/μL	Ca	8.8	mg/dL			
			Na	129	mEq/L	Tumor markers		
			K	4.3	mEq/L	CEA	1.4	ng/mL
			Cl	97	mEq/L	SCC	0.6	ng/mL
			Glu	305	mg/dL	CYFRA	3.3	ng/mL
			HbA1c	9.1	%	pro‐GRP	29.4	pg/mL

Alb, albumin; ALT, alanine transaminase; AST aspartate aminotransferase; BUN, blood urea nitrogen; Ca, calcium; Cl, chloride; CRE, creatinine; Eos, eosinophils; Glu, glucose; Hb, hemoglobin; HbA1c, glycated hemoglobin; Ht, hematocrit; K, potassium; LDH, lactate dehydrogenase; Lym, lymphocytes; Na, sodium; Neut, neutrophils; PLT, platelets; RBC, red blood cells; WBC, white blood cells; y‐GTP, gamma glutamyl transpeptidase.

**Figure 2:**
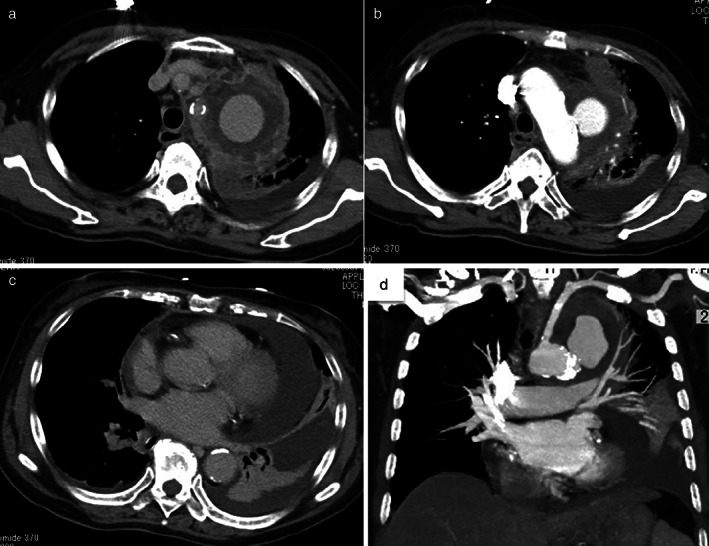
Results of urgent contrast‐enhanced computed tomography (CT) of the chest. (**a**–**c**) Axial images. (**d**) Sagittal images. Contrast‐enhanced CT of the chest reveals pseudoaneurysm of the descending thoracic aorta. Pleural effusion and pericardial fluid are worse than those from CT of the chest on admission.

Further examination was planned, but the patient's respiratory failure suddenly worsened (oxygen saturation, 90%; on O_2_ at 4 L/minute via nasal cannula) on hospital day 2. Urgent contrast‐enhanced CT of the chest (Fig 2) revealed a vascular rupture site on the left wall of the descending aorta, which showed contrast medium leaking out of a blood vessel. A ruptured PTA was therefore diagnosed. Emergency thoracic endovascular aortic repair (TEVAR) was performed the same day. Procalcitonin (PCT) level was 2.74 ng/mL, and methicillin‐sensitive *Staphylococcus aureus* (MSSA) was detected from both sputum and blood cultures. A mycotic thoracic aortic aneurysm was therefore diagnosed, and meropenem was administered at 1 g/day postoperatively.

**Figure 3:**
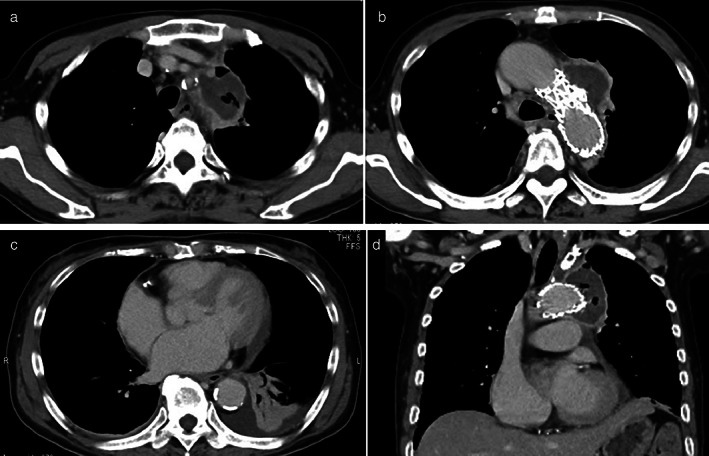
Contrast‐enhanced CT after thoracic endovascular aortic repair (TEVAR). (**a**–**c**) Axial images. (**d**) Sagittal images. No stent migration or endoleak is observed, and the aneurysm is reduced.

Contrast‐enhanced CT (Fig 3) performed two months after TEVAR showed a decrease in the size of the aneurysm. The clinical course was good, and the patient was discharged on hospital day 56.

## Discussion

Pseudoaneurysm is a condition in which one layer or complete aortic wall rupture is present, but progressive bleeding does not occur due to the remaining wall or surrounding mediastinal structures.^4^

PTA is a rare complication of aortic surgery that occurs in less than 0.5% of cardiac surgery cases. PTA may also occur secondarily after infection or trauma, but its exact frequency remains unclear.^5^, ^6^, ^7^

As a condition similar to that of pseudoaneurysm, Jones *et al*. reported a case in which hematoma caused by rupture of an aneurysm was contained by the surrounding tissue and showed stable hemodynamics without progressive bleeding as “contained rupture”.^8^

About 80% of patients with contained rupture of a thoracic aortic aneurysm (in other words, PTA) reportedly die within 24 hours due to rerupture.^9^ Clinicians thus need to diagnose PTA quickly and accurately.

The imaging features of pseudoaneurysm on plain CT are high or low attenuation suggestive of hematoma surrounding the aneurysm, and the boundary with the aneurysm is often unclear.^10^, ^11^, ^12^ Plain CT scan of the chest in this case also showed a mass of aneurysms and surrounding infiltrative shadows. Image findings mimicking locally advanced lung cancer were therefore exhibited.

The characteristics of imaging findings for plain CT of ruptured or about‐to‐rupture aortic aneurysm include a linear hyperattenuated area around the aneurysm, attributed to fresh hematoma, called a high‐attenuating crescent sign.^13^ Likewise in this case, imaging findings that seemed to represent a high‐attenuating crescent sign were confirmed inside the tumor. We consider that we should have performed contrast‐enhanced CT at the first visit.

In addition, the patient was diagnosed with mycotic aortic aneurysm (MAA) caused by MSSA. MAAs are rare aortic aneurysms caused by infection. This disease shows a poor prognosis, accounting for 0.5%–1.3% of all aortic aneurysms and a hospital mortality rate of 23.5%–37%.^14^, ^15^ A number of routes account for infection of the arterial walls, including septic emboli, bacteremic seeding of an existing intimal injury or atherosclerotic plaque.^16^ In this case, physical examination by a dentist revealed dental caries necessitating tooth extraction. The patient was therefore diagnosed with MSSA sepsis through the gingival sulcus as the point of entry. *S. aureus* also tends to form abscesses in various organs, as well as sepsis.^17^

The patient had advanced arteriosclerosis due to pre‐existing diabetes and hypertension. As a result, MAA was presumed to have developed due to the formation of an abscess by MSSA in the weakened descending aorta.

MAAs are prone to rapid aneurysm expansion due to weakening of the arterial wall, and the risk of rupture can be 85% or more.^11^, ^14^ In addition, MAA has been reported to often cause contained rupture.^11^, ^18^

The patient in this report was also diagnosed with a contained rupture, similar to the reports above.^11^, ^18^ Aneurysm rupture due to rapid expansion of the aneurysm is also possible.

Although the patient had a high PCT level of 2.74 ng/mL, PCT has been used as an important marker for bacterial infection and has been reported as useful in differentiating infection from tumorigenic fever.^19^

Since MAA causes contained rupture and hemodynamics remain stable in some cases at the first visit, use of PCT as a diagnostic aid appears desirable to distinguish bacterial infection from mediastinal tumor.

In conclusion, PTA should be included among the differential diagnoses of mediastinal tumor. To assist in the differential diagnoses, clinicians need to be familiar with the imaging findings of PTA.

## Disclosure

The authors have no conflicts of interest to declare.
